# Relative Frequency of Paradoxical Growth and Trailing Effect with Caspofungin, Micafungin, Anidulafungin, and the Novel Echinocandin Rezafungin against *Candida* Species

**DOI:** 10.3390/jof6030136

**Published:** 2020-08-17

**Authors:** Zoltán Tóth, Lajos Forgács, Tamás Kardos, Renátó Kovács, Jeffrey B. Locke, Gábor Kardos, Fruzsina Nagy, Andrew M. Borman, Awid Adnan, László Majoros

**Affiliations:** 1 Department of Medical Microbiology, Faculty of Medicine, University of Debrecen, 4032 Debrecen, Hungary; toth.zoltan@med.unideb.hu (Z.T.); forgacs.lajos.89@gmail.com (L.F.); kovacs.renato@med.unideb.hu (R.K.); kg@med.unideb.hu (G.K.); nagyfruzsina0429@gmail.com (F.N.); mirilmedic@gmail.com (A.A.); 2 Doctoral School of Pharmaceutical Sciences, University of Debrecen, 4032 Debrecen, Hungary; 3 Department of Pulmonology, Faculty of Medicine, University of Debrecen, 4032 Debrecen, Hungary; kardos.tamas@med.unideb.hu; 4 Faculty of Pharmacy, University of Debrecen, 4032 Debrecen, Hungary; 5 Cidara Therapeutics, Inc., 6310 Nancy Ridge Dr., Suite 101, San Diego, CA 92121, USA; jlocke@cidara.com; 6 UK National Mycology Reference Laboratory, Public Health England, Science Quarter, Southmead Hospital, Bristol BS10 5NB, UK; andy.borman@nbt.nhs.uk; 7 Medical Research Council Centre for Medical Mycology (MRC CMM), University of Exeter, Exeter EX4 4QD, UK

**Keywords:** rezafungin, trailing effect, paradoxical growth, *Candida*, echinocandin, *C. auris*

## Abstract

Rezafungin is a next-generation echinocandin that has favorable pharmacokinetic properties. We compared the occurrence of paradoxical growth (PG) and trailing effect (TE) characteristics to echinocadins with rezafungin, caspofungin, micafungin and anidulafungin using 365 clinical *Candida* isolates belonging to 13 species. MICs were determined by BMD method according to CLSI (M27 Ed4). Disconnected growth (PG plus TE) was most frequent with caspofungin (49.6%), followed by anidulafungin (33.7%), micafungin (25.7%), while it was least frequent with rezafungin (16.9%). PG was relatively common in the case of caspofungin (30.1%) but was rare in the case of rezafungin (3.0%). *C. tropicalis*, *C. albicans*, *C. orthopsilosis* and *C. inconspicua* exhibited PG most frequently with caspofungin, micafungin or anidulafungin. PG never occurred in the case of *C. krusei* isolates. Against *C. tropicalis* and *C. albicans*, echinocandins frequently showed PG after 24 h followed by TE after 48 h. All four echinocandins exhibited TE for the majority of *C. auris* and *C. dubliniensis* isolates. Disconnected growth was common among *Candida* species and was echinocandin- and species-dependent. In contrast to earlier echinocandins, PG was infrequently found with rezafungin.

## 1. Introduction

Currently, echinocandins (anidulafungin, caspofungin and micafungin) are the first-line antifungals for the treatment of invasive *Candida* infections [1]. Infections of normally sterile body sites often require higher echinocandin exposure to eliminate the fungus [1,2,3,4,5]. Higher echinocandin concentrations have been shown to induce a variety of stress adaptation pathways and increased cell wall chitin in vitro [6,7,8] in response to depletion of cell wall glucan, which allow the fungus to grow at high antifungal concentrations (8–64 mg/L) and has been termed Eagle effect or paradoxical growth (PG) effect [9,10,11,12,13].

Another peculiar growth phenomenon with echinocandins against *Candida* species is the trailing effect (TE), where complete growth inhibition is not achieved or occurs at dilutions well above the 50% endpoint used to determine broth MIC values [11,12,13]. Echinocandin-induced TE was found in cases of *C. dubliniensis* (10–80%), *C. tropicalis* (17.0–26.4%) and *C. guilliermondii* (7.7–15.4%) in various studies, but TE was very rare or lacking in the case of other *Candida* species [11,12,13]. The caspofungin-induced TE, at least in the case of *C. dubliniensis,* proved to be a fungistatic effect as determined by time-kill methodology [14]. PG and TE are collectively termed as disconnected growth, and can cause mistakes in interpretation of MIC determination, may impact other in vitro assays, thus may cause problems during susceptibility testing. High level of TE, for example, may be misinterpreted as resistance. However, the clinical relevance of these effects observed in vitro has not been substantiated [13,15,16].

Rezafungin is a next-generation echinocandin with excellent in vitro activity comparable to the three licensed echinocandins against common as well as rare *Candida* species [17]. Rezafungin attains high concentrations in vivo, exceeding those measured for the three approved echinocandins, due to its long half-life and front-loaded dosing regimen, leading to higher drug exposure in blood and tissues [1,7,17,18] Given the distinction of higher drug concentrations for rezafungin relative to the currently approved echinocandins and that rezafungin PG and TE trends have not yet been characterized, this study compares the frequency of PG and TE for rezafungin with that for caspofungin, micafungin, and anidulafungin against clinically important *Candida* species in parallel experiments.

## 2. Materials and Methods

A panel of 349 non-duplicate clinical isolates of 12 *Candida* species collected in Hungary from normally sterile body sites and 16 *C. auris* isolates were tested (Table 1). All isolates, including *C. auris* isolates, were the same as in our previous study [17]. Out of the 16 *C. auris* isolates, eight belonged to the South Asian clade, six to the South African clade and one isolate, together with the type strain NCPF 13029 belonged to the East Asian clade. MICs were determined by BMD method according to CLSI (M27 Ed4) in RPMI-1640 [19]. We used tissue culture–treated microtiter test plates (TPP Techno Plastic Products AG, Trasadingen, Switzerland, cat. # 92097). Rezafungin pure powder was provided by Cidara Therapeutics (lot # C15071064-CF16001). Caspofungin (cat. # CSF00A-100; lot # 160130), micafungin (cat. # MCF00N; lot # 170822) and anidulafungin (cat. # ADF00-100; lot # 170218) were obtained from Molcan Corporation, Canada. Echinocandins were dissolved in 100% DMSO and diluted further in RPMI-1640. Concentration ranges were 0.06–32 mg/L for all four echinocandins. This range is higher than the range used in the previous susceptibility study to enhance detection of PG and TE [17]. At the corresponding concentrations, all MICs were the same within one dilution as the MICs determined in the previous study. Drug-free and yeast-free controls were also included. CLSI-approved quality control strains (*C. parapsilosis* ATCC 22019 and *C. krusei* ATCC 6258) and other ATCC and type strains are shown in Table 2. MICs were determined at least twice.

MICs were read visually after 24 h according to the partial inhibition criterion; PG and TE were evaluated both after 24 and 48 h. PG was defined as visible growth occurring at higher but not at lower supra-MIC concentrations [9,14]. TE was defined as when yeasts show reduced but observable growth without magnification in all wells at supra-MIC concentrations [11,14].

In order to better visualize the PG and TE, MICs were also determined on agar media via MIC Test Strip (MTS; Liofilchem) with ten selected isolates (including the ATCC and type strains) each of *C. albicans*, *C. dubliniensis*, *C. tropicalis*, *C. krusei*, *C. auris* and *C. inconspicua*. As caspofungin susceptibility testing is not recommended due to the observed significant interlaboratory variability and a rezafungin gradient strip-based MIC device is not yet available, we used anidulafungin and micafungin MTSs. MTS MICs were carried out according to the instructions of the manufacturer (using inoculum suspension of ~10^6^ cells/mL), and the results were read after 24 and 48 h [3].

## 3. Results

Table 1 shows the MIC distribution and the most frequent concentrations at which PG was observed. Table 2 shows the PG and TE for the ATCC and type strains after 24 and 48 h.

Rezafungin induced PG with the lowest cumulative frequency (3.0%) compared to caspofungin (30.1%), anidulafungin (19.5%), and micafungin (15.3%) after 48 h. The lowest concentrations of caspofungin, micafungin, anidulafungin, and rezafungin at which PG occurred were 2, 0.5, 0.5, and 2 mg/L, respectively. The cumulative frequency of TE was markedly lower with micafungin (10.4%), rezafungin (13.9%) and anidulafungin (14.2%) compared to caspofungin (19.5%).

**Figure 1:**
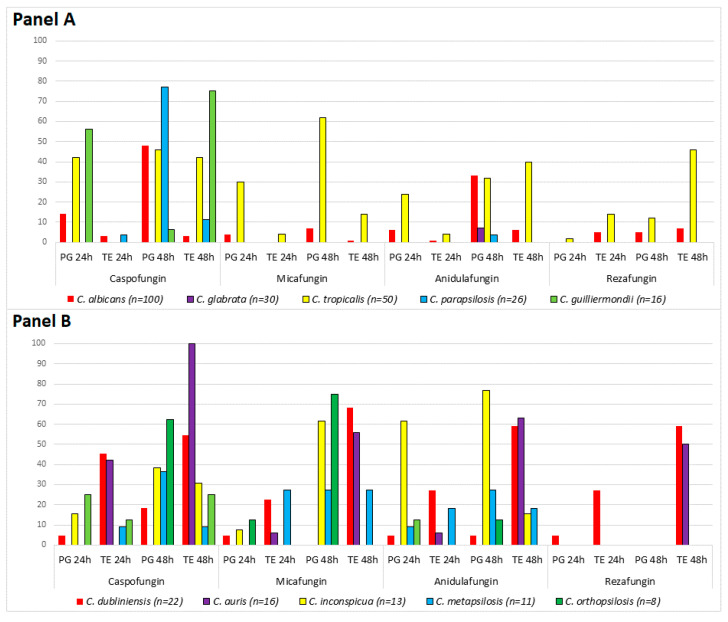
Frequency (%) of paradoxical growth (PG) and trailing effect (TE) among Candida albicans, C. glabrata, C. tropicalis, C. parapsilosis, C. guilliermondii (**Panel A**) and C. dubliniensis, C. auris, C. inconspicua, C. metapsilosis and C. orthopsilosis (**Panel B**).

PG occurring after 24 h followed by TE after 48 h was observed with all four drugs in case of *C. albicans*, *C. dubliniensis* and *C. tropicalis* (in 6, 1 and 13 isolates with caspofungin; in 5, 1 and 3 isolates with anidulafungin; in 1, 1 and 2 isolates with micafungin; and in 1, 1 and 1 isolates with rezafungin, respectively). A similar phenomenon was observed for 9 isolates of *C. guilliermondii* with caspofungin and for one isolate of *C. inconspicua* with anidulafungin and caspofungin (Figure 1). The same phenomenon was observed in cases of *C. dubliniensis* CD36, *C. auris* NCPF 13029 = CBS 10913 and *C. inconspicua* ATTC 16,783 type strains with rezafungin, anidulafungin and caspofungin (Table 2). Among *C. tropicalis* and *C. albicans* isolates showing PG with anidulafungin, caspofungin and micafungin both after 24 and 48 h, the concentrations at which PG was observed after 48 h were lower than those at which PG was observed after 24 h. For example, after 24 h the most frequent echinocandin concentration range where PG started was 16–32 mg/L, but after 48 h PG frequently started at 1–2 mg/L (range 1–32 mg/L). This phenomenon was observed in cases of *C. tropicalis* ATCC 750 with caspofungin and *C. inconspicua* ATCC 16783 with anidulafungin (Table 2).

In *C. albicans*, PG was uncommon with micafungin (3%) or rezafungin (7%) after 48 h (Figure 1), and TE was low (1–7%) for all four echinocandins. The frequency of PG was low (0–18.2%) for *C. dubliniensis*, but 54.5 to 68.2% of isolates showed TE with all echinocandins after 48 h (Figure 1). In *C. tropicalis*, PG and TE at 48 h was the lowest with rezafungin (12%) and micafungin (14%), respectively (Figure 1).

In contrast to Chamilos et al., neither PG nor TE were observed for *C. krusei* with the three licensed echinocandins, nor were either phenomena observed in this study with rezafungin [9]. The frequency of PG and TE with anidulafungin, caspofungin and micafungin was low in cases of *C. glabrata*, *C. kefyr*, and *C. lusitaniae* (Figure 1) and were completely absent with rezafungin. PG was never observed for *C. auris* clinical isolates with the exception of the type strain with anidulafungin and caspofungin after 24 h, but 50–100% of our isolates including the type strain exhibited strong TE with all four echinocandins after 48 h (Figure 1 and Table 2).

Neither PG nor TE were observed with rezafungin for “psilosis” group species and *C. inconspicua*. PG for *C. parapsilosis sensu stricto*, *C. orthopsilosis*, and *C. metapsilosis* was frequently found with caspofungin, and also with micafungin for *C. orthopsilosis* (Figure 1). For *C. inconspicua*, the frequency of PG with micafungin and anidulafungin (61.6 and 76.9%, respectively) were markedly higher than with caspofungin (38.5%).

**Figure 2:**
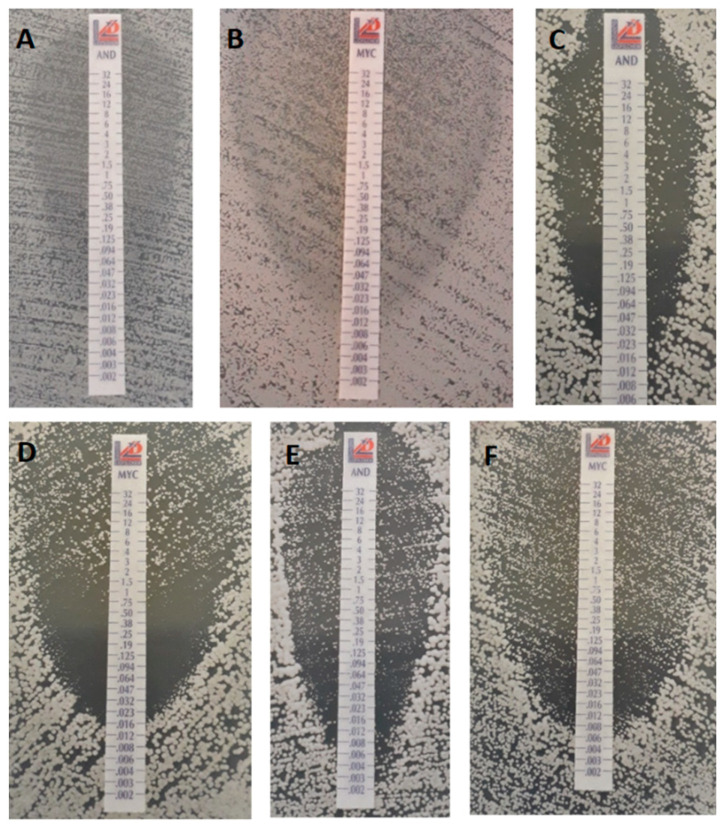
Trailing effect (TE) and paradoxical growth (PG) using anidulafungin (AND) and micafungin (MYC) MIC Test Strips (MTSs) against *Candida* species. For *C. auris*, TE was observed both after 24 (**A**) and 48 (**B**) hours with anidulafungin and micafungin MTS while *C. tropicalis* showed TE (**C**) and PG (**D**) after 48 h, respectively. In the case of *C. dubliniensis*, TE was observed after 48 h with anidulafungin (**E**) and micafungin (**F**).

MTS results with anidulafungin and micafungin showed identical results with the BMD test with the exception of *C. inconspicua*. We observed either clear growth inhibition zones or well-defined visible growth at higher concentrations (PG) or dense growth throughout the elliptic growth inhibition zones (TE) with the tested *C. albicans*, *C. tropicalis* strains after 24 or 48 h (Figure 2). In the case of *C. dubliniensis*, a clear growth inhibition zone was noticed after 24 h with both echinocandins, but after 48 h TE was found with both drugs in 5 of 10 clinical isolates and with the CD36 type strain (Figure 2). *C. auris* exhibited TE even after 24 h (4 of 10 isolates with both drugs); after 48 h, all tested isolates showed TE with anidulafungin and micafungin (Figure 2). In cases of *C. krusei* and *C. inconspicua,* clear growth inhibition zones were observed in all cases.

## 4. Discussion

Paradoxical growth with the three licensed echinocandins has been detected with *C. tropicalis*, *C. albicans*, *C. parapsilosis*, *C. krusei, C. dubliniensis* and *C. auris* using both CLSI and EUCAST BMD methods [9,10,11,12,13,20]. These previous studies showed that PG is echinocandin-, species-, isolate- and medium-dependent. However, the frequency of PG was evaluated under highly variable conditions. For example, EUCAST uses a 100-fold higher starting inoculum compared to CLSI and spectrophotometric reading, and the incubation time used by different researchers varied from 24 to 120 h. Moreover, variation in the quality of microtiter plates used in the BMD tests may impact assessment of the PG frequency across studies [9,10,11,12,13,17,20]. Data about echinocandin-induced TE against *Candida* species are scant, in part because MIC values have been read after 24 h, as recommended by both CLSI and EUCAST methods for more than 10 years, and TE is typically observed after 48 h [11,13,19].

In the present study, in line with others, caspofungin was most frequently associated with PG in both common (*C. albicans*, *C. tropicalis* and *C. parapsilosis)* and rare (*C. orthopsilosis*, *C. metapsilosis* and *C. inconspicua*) *Candida* spp. The frequency of PG with micafungin was high in *C. tropicalis*, *C. orthopsilosis* and *C. inconspicua*. Interestingly, although rezafungin is closely related structurally to anidulafungin (rezafungin has a choline moiety at the C5 ornithine position), only 2 of 13 *Candida* species (*C. albicans* and *C. tropicalis*) showed PG with rezafungin compared with 7 of 13 species with anidulafungin [18,21].

Previous studies showed that the outcomes of invasive *Candida* infections treated with elevated daily doses of caspofungin or micafungin are numerically, though not statistically, less favorable compared to standard daily doses, suggesting the possible role of PG in vivo [15,16]. Similar results were obtained by Rueda et al. in 117 candidemic patients initially treated with standard daily doses of the three licensed echinocandins. Interestingly, 60.4–69.8% and 17.0–26.4% of the *C. tropicalis* isolates studied showed PG and TE, respectively, with anidulafungin, caspofungin and micafungin [13]. In our study, the rates of disconnected growth (PG plus TE) for *C. tropicalis* were similar with anidulafungin, caspofungin and micafungin (72–88%) and the lowest with rezafungin (58%).

It is noteworthy that PG was low (0–18.2%) or absent with *C. dubliniensis* and *C. auris* isolates, respectively, but 54.5–68.2% of *C. dubliniensis* and 50–100% of *C. auris* isolates showed TE to all four echinocandins after 48 h. Our results show partial concordance with Kordalewska et al., who found 100% PG and 100% TE with caspofungin against *C. auris* isolates after 24 and 48 h, respectively, but neither with anidulafungin nor with micafungin. Moreover, they observed difficulties in reading the MICs [20]. In another study, anidulafungin and caspofungin were found to be fungistatic against *C. auris* [22]. TE observed among *C. auris* and *C. dubliniensis* isolates may have played a role in clinical failures of echinocandin therapy reported against these less common *Candida* species [23,24,25].

Our data suggest a correlation between PG and TE. First, numerous *C. tropicalis*, *C. albicans*, *C. dubliniensis* and *C. inconspicua* isolates showed growth only at higher echinocandin concentrations after 24 h (PG), but these isolates grew in all of the wells above the MIC producing prominent TE after 48 h. Second, in the cases of *C. tropicalis* and *C. albicans* isolates showing PG both after 24 and 48 h, the number of clear wells above MIC was reduced from 6–7 to 2–3, respectively, indicating that, with longer incubation times, visible re-growth started at lower echinocandin concentrations. These effects may be the consequence of the echinocandin-induced chitin synthesis at wide concentration ranges (0.25–16 mg/L) as observed by Rueda et al. with caspofungin against *C. albicans*, reporting visually observable re-growth after 48 h [8]. The relevance of increased chitin content at lower caspofungin concentrations but above the MIC was demonstrated in our previous work, when adding the chitin synthesis inhibitor nikkomycin Z to caspofungin, even at sub-MIC concentrations 0.12 or 1 mg/L, increased killing against *C. albicans* and *C. tropicalis* (synergistic effect) [26].

## 5. Conclusions

This study is the first to report PG and TE trends for rezafungin and helps to inform future in vitro work with this novel echinocandin. PG and TE observed among *Candida* species using the CLSI BMD method were echinocandin-, species-, and isolate-dependent. Our BMD results were also supported by MTS results with anidulafungin and micafungin. Rezafungin induced PG plus TE with the lowest frequency among the four echinocandin agents tested. PG with caspofungin, micafungin, or anidulafungin occurred most frequently for *C. tropicalis*, *C. albicans*, *C. orthopsilosis, C. metapsilosis,* and *C. inconspicua*, but never for *C. krusei*. The high frequency of TE observed among *C. tropicalis*, *C. auris* and *C. dubliniensis* isolates may have played a role in clinical failures of echinocandin therapy reported against these common and less common *Candida* species. Therefore, while the clinical relevance of disconnected growth phenomena such as PG remains questionable as suggested in preclinical models [2,3,27], the possible relationship between PG and TE observed in vitro warrants consideration.

## Figures and Tables

**Table 1:** MIC distributions of echinocandins for the 13 *Candida* species. MICs were read after 24 h using partial inhibition criterion. Grey cells mark the most frequent concentration ranges where paradoxical growth (PG) was noticed.

Species (*n*)	Drug	Number of Isolates Inhibited at Each MIC Value (µg/mL)									
		≤0.06	0.12	0.25	0.5	1	2	4	8	16	32
***C. albicans*** **(100)**	**RZF**	100									
	**ANF**	100									
	**CSF**	9	5	52	34						
	**MCF**	99	1								
***C. glabrata*** **(30)**	**RZF**	18	12								
	**ANF**	30									
	**CSF**		1	11	18						
	**MCF**	30									
***C. parapsilosis s*** ***ensu stricto*** **(26)**	**RZF**				5	11	10				
	**ANF**				4	10	12				
	**CSF**				4	16	6				
	**MCF**				3	14	9				
***C. tropicalis*** **(50)**	**RZF**	50									
	**ANF**	50									
	**CSF**	2	9	24	15						
	**MCF**	48	2								
***C. krusei*** **(30)**	**RZF**	16	14								
	**ANF**	27	2	1							
	**CSF**			2	6	22					
	**MCF**		9	21							
***C. kefyr*** **(16)**	**RZF**	10	6								
	**ANF**	15	1								
	**CSF**			12	4						
	**MCF**	11	5								
***C. lusitaniae*** **(27)**	**RZF**	3	17	7							
	**ANF**	26	1								
	**CSF**		1	4	12	10					
	**MCF**	9	11	6	1						
***C. guilliermondii*** **(16)**	**RZF**				3	12	1				
	**ANF**				3	11	2				
	**CSF**			1	9	6					
	**MCF**				7	7	2				
***C. dubliniensis*** **(22)**	**RZF**	22									
	**ANF**	22									
	**CSF**	2	8	10	2						
	**MCF**	22									
***C. auris*** **(16)**	**RZF**	4	6	6							
	**ANF**	9	4	2	1						
	**CSF**			4	7	5					
	**MCF**		6	10							
***C. orthopsilosis*** **(8)**	**RZF**			3	2	3					
	**ANF**		1	1	3	3					
	**CSF**				3	5					
	**MCF**			1	4	3					
***C. metapsilosis*** **(11)**	**RZF**			2	9						
	**ANF**		4	5	2						
	**CSF**		1	5	5						
	**MCF**		1	6	4						
***C. inconspicua*** **(13)**	**RZF**	13									
	**ANF**	13									
	**CSF**	2	4	7							
	**MCF**	13									

**Table 2:** Pattern of paradoxical growth (PG) or trailing effect (TE) with rezafungin, anidulafungin, caspofungin and micafungin against *Candida* ATCC and type strains after 24 and 48 h. Concentration ranges (mg/L) indicate where PG was noticed.

	Rezafungin	Anidulafungin			Caspofungin		Micafungin	
	24 h	48 h	24 h	48 h	24 h	48 h	24 h	48 h
***C. krusei* ATCC 6258**	-	-	-	-	-	-	-	-
***C. parapsilosis* ATCC 22019**	-	-	-	PG (8–32)	-	-	-	-
***C. albicans* ATCC 10231**	-	-	-	-	-	PG (16–32)	-	-
***C. glabrata* ATCC 90030**	-	-	-	PG (16–32)	-	-	-	-
***C. tropicalis* ATCC 750**	-	-	-	-	PG (16–32)	PG (4–32)	-	-
***C. orthopsilosis* ATCC 96139**	-	-	-	-	PG (8)	PG (8)	PG (8)	PG (8)
***C. metapsilosis* ATCC 96144**	-	-	-	-	-	PG (8)	-	-
***C. guilliermondii* ATCC 6260**	-	-	-	-	-	PG (32)	-	-
***C. dubliniensis* CD36**	PG (16–32)	TE	TE	TE	TE	TE	-	-
***C. auris* NCPF 13029 = CBS 10913 (type strain)**	-	TE	PG (32)	TE	PG (8–32)	TE	-	-
***C. inconspicua* ATCC 16783**	-	-	PG (8–32)	PG (1–32)	PG (4–8)	TE	-	PG (8–32)
